# Strategies to Overcome PD-1/PD-L1 Blockade Resistance: Focusing on Combination with Immune Checkpoint Blockades

**DOI:** 10.7150/jca.108163

**Published:** 2025-07-24

**Authors:** Dinglin Liu, Haoyue Xiao, Ying Xiang, Dian Zhong, Yuchen Liu, Yunfei Wang, Weijia Zhang

**Affiliations:** 1 Department of Oncology, First Affiliated Hospital of Yangtze University, Jingzhou, Hubei 434023, China.; 2 Laboratory of Oncology, Center for Molecular Medicine, School of Basic Medicine, Health Science Center, Yangtze University, 1 Nanhuan Road, Jingzhou, Hubei 434023, China.

**Keywords:** PD-1, PD-L1, immune checkpoint blockade, resistance, combination therapy

## Abstract

In recent years, immune checkpoint blockades (ICBs) have made rapid progress in the field of cancer treatment, providing significant therapeutic effects and survival benefits, especially in patients with advanced refractory tumors. PD-1/PD-L1 blockade is one of the most widely used ICBs. However, its application is limited by low response rate and drug resistance. It is of great significance to investigate the complex mechanisms of PD-1/PD-L1 blockade resistance. In this review, we outline some crucial aspects, including lack of effector T cells, lack of target PD-1/PD-L1, poor immunogenicity of tumors, immunosuppressive TME, and other mechanisms (such as metabolism, epigenetic alterations, and gut microbiota). Combination therapy has become a promising strategy to overcome drug resistance. Based on the upregulation of other immune checkpoints after PD-1/PD-L1 blockade treatment, we focus on the combination with other ICBs, including CTLA-4, TIM-3, LAG-3, TIGIT, VISTA, and some emerging immune checkpoints, so as to provide evidence for improving the benefit of ICBs in cancers.

## Introduction

For a long time, various strategies have been employed to boost the immune response to fight against cancer. However, the frequent adverse effects associated with these treatments highlight their limitations. Notably, over the past decade, the blockade of programmed death-1 (PD-1)/programmed death-ligand 1 (PD-L1) has transcended the limitations of previous cancer immunotherapy 1. Specifically, this innovative treatment is capable of defending against tumors by restoring the innate anti-tumor immune response with a minor or no increase in adverse effects, a concept described as “Normalized Cancer Immunotherapy” 2. Mechanistically, malignant tumors may have the ability to stimulate the expression of various immune checkpoints, such as PD-1 and PD-L1, thereby inhibiting the normal activation of T cells within the tumor microenvironment (TME), which ultimately allows the tumor to escape immune attack. Through reactivating immune cells, immune checkpoint therapy can achieve immune normalization without excessively amplifying the immune response, thereby reducing the occurrence of severe toxic effects 3.

The first antibody against PD-1 (nivolumab) was approved by the FDA in 2014. Since then, blocking antibodies against PD-1 or PD-L1 (anti-PD-(L)1) have been approved for application in multiple tumors. Additionally, clinical trials have demonstrated that PD-1/PD-L1 blockade can provide a durable clinical response in specific tumor types and patient populations and may lead to long-term tumor non-progression after treatment discontinuation in some patients, thereby potentially enhancing overall survival 4, 5. However, the efficacy is confined to a small portion of individuals. Many patients quickly acquire resistance and experience different immune-related adverse events to some extent 6, 7. Furthermore, the lack of effective biomarkers makes it challenging to predict which patients will benefit from anti-PD-1/PD-L1 treatment 8. Additionally, the human immune system is a dynamic environment, contributing to the considerable challenges of immunotherapy 9. Even if there are many challenges, on the whole, for patients with late-stage malignant tumors that have undergone extensive metastasis or are unresponsive to traditional anti-cancer therapies (radiation, chemotherapy, surgery, targeted therapies), immunotherapy provides a promising and innovative option.

Considering the low response rate to monotherapy and the issue of resistance, some experts propose that the combinations of multiple immune checkpoint blockades (ICBs) might expand the group of patients benefiting from treatment, lengthen the objective response rate, and reduce the occurrence of resistance. This has been supported by the findings from multiple preclinical and clinical trials 10. The current review delves into the molecular mechanisms of PD-1/PD-L1 blockade resistance, emphasizing the synergistic anti-tumor effects of anti-PD-1/PD-L1 combined with other ICBs, so as to provide evidence for improving the benefits of ICBs in cancers.

## PD-1 Pathway and Clinical Applications in Cancer Therapy

### Structure and function of the PD-1 pathway

PD-1 (CD279) was discovered by Tasuku Honjo and his team in 1992, who found that PD-1 levels increased during a classic type of programmed cell death process in mouse T cell hybridomas 11. Later on, PD-1 was recognized as an immune checkpoint that not only negatively regulated the peripheral immune response but also participated in maintaining immune tolerance 12. Structurally, PD-1 belongs to the CD28 family and is a transmembrane glycoprotein with the extracellular domain, the transmembrane segment, and the cytoplasmic tail domain. The cytoplasmic tail domain contains two motifs: an immune receptor tyrosine-based inhibitory motif (ITIM) and an immune receptor inhibitory tyrosine-based switch motif (ITSM). The ITSM motif is the key structure that mediates the suppression of immune response 13.

PD-1 pathway not only includes PD-1 but also its ligands, PD-L1 (B7-H1, CD274) and PD-L2 (B7-DC, CD273). Among these, PD-L1, as the primary ligand for PD-1, is commonly upregulated in tumor cells and also expressed in B cells, T cells, dendritic cells, macrophages, bone marrow-derived mast cells, and some non-immune cells 14. In contrast, PD-L2, as the secondary ligand for PD-1, is more confined in antigen-presenting cells (APCs, for example, macrophages and dendritic cells) stimulated by cytokines, and can also be induced in other immune cells, non-immune cells, and tumor cells 15. Mechanistically, the interaction between PD-1 and its ligands is mainly mediated by the tyrosine phosphatase SHP-2, which dephosphorylates signaling molecules downstream of the T-cell receptor (TCR), thereby blocking the activation of T-cell activation 1. Furthermore, PI3K/Akt/mTOR signaling pathway and RAS/MEK/ERK signaling pathway are the main downstream of PD-1 and its ligands, both of which are associated with the decreased T cell function and immunosuppression 16.

### Cancer immune evasion via PD-1/PD-L1

PD-1/PD-L1 pathway, involved in protecting cells against T cell attack, is considered to be one of the major mechanisms of tumor immune escape. The PD-L1 expression levels in various types of cancers have been confirmed to be correlated with negative outcomes 17, 18. Mechanistically, tumor cells upregulate the expression of PD-L1, and subsequently, the overexpressed PD-L1 binds to and transmits inhibitory signals to PD-1-expressing T cells, especially CD8^+^ T cells, thus evading the attack of the immune system 19.

The expression of PD-L1 in tumors is mainly regulated by inflammatory mediators, and interferon (IFN)-γ is a notable one. Paradoxically, to fight tumor cells, anti-tumor immune cells secrete IFN-γ, but IFN-γ, in turn, induces the expression of genes (such as PD-L1) involved in tumor immune evasion 20. The natural expression of the PD-L1 protein is limited to specific cancer tissues, which is induced by IFN-γ in the TME 21. However, when tumor cells that do not naturally express PD-L1 protein are treated with IFN-γ, most of them are induced to express PD-L1 protein 22. PD-L1 expression is negative in the majority of cancer cell lines cultured *in vitro*. When melanoma cells were implanted into IFN-γ-deficient mice, the unsuccessful upregulation of PD-L1 demonstrated that IFN-γ was required for PD-L1-induced expression in tumor cells 23. On the one hand, IFN-γ can upregulate the expression of major histocompatibility complex (MHC)-I and promote T cell differentiation, thereby enhancing anti-tumor immune response. On the other hand, induced PD-L1 expression by IFN-γ also helps tumors achieve immune evasion by binding with the PD-1 on T cells 21.

### Cancer immunotherapy with PD-1/PD-L1 blockade

To date, many anti-PD-1 antibodies (Abs) and anti-PD-L1 Abs have been developed to block PD-1/PD-L1 signaling. Anti-PD-1 Abs (nivolumab, pembrolizumab, and cemiplimab) and anti-PD-L1 antibodies (atezolizumab, avelumab, and durvalumab) have been approved by FDA for some solid tumor and hematologic cancers 24. Some clinical trials have confirmed the anti-tumor efficacy of PD-1/PD-L1 blocking therapies and have consistently demonstrated clinical therapeutic benefits across a wide range of cancer types 25-27. Mechanistically, blocking PD-1/PD-L1 led to increased proliferation of CD8^+^ T cells 28. Another research showed that the reinvigoration of CD8^+^ T cells after anti-PD-1 therapy was associated with clinical outcomes 29. Patients with tumor-infiltrating CD8^+^ T cells exhibited a greater response to anti-PD-1 treatment 30. PD-1 blocking therapy could increase CD8^+^ T cells in the peripheral blood of patients with non-small cell lung cancer (NSCLC) 31. PD-1 blocking could not only strengthen the activity of T cells that target cancer cells but also boost the activity of other immune cells in the TME, such as NK cells and B cells 32.

However, researchers and clinicians also pay attention to the immune-related adverse events associated with checkpoint blockade treatment and strive to balance the risks and benefits 33.

### Immune-related adverse events associated with PD-1/PD-L1 blockade

**Figure 1:**
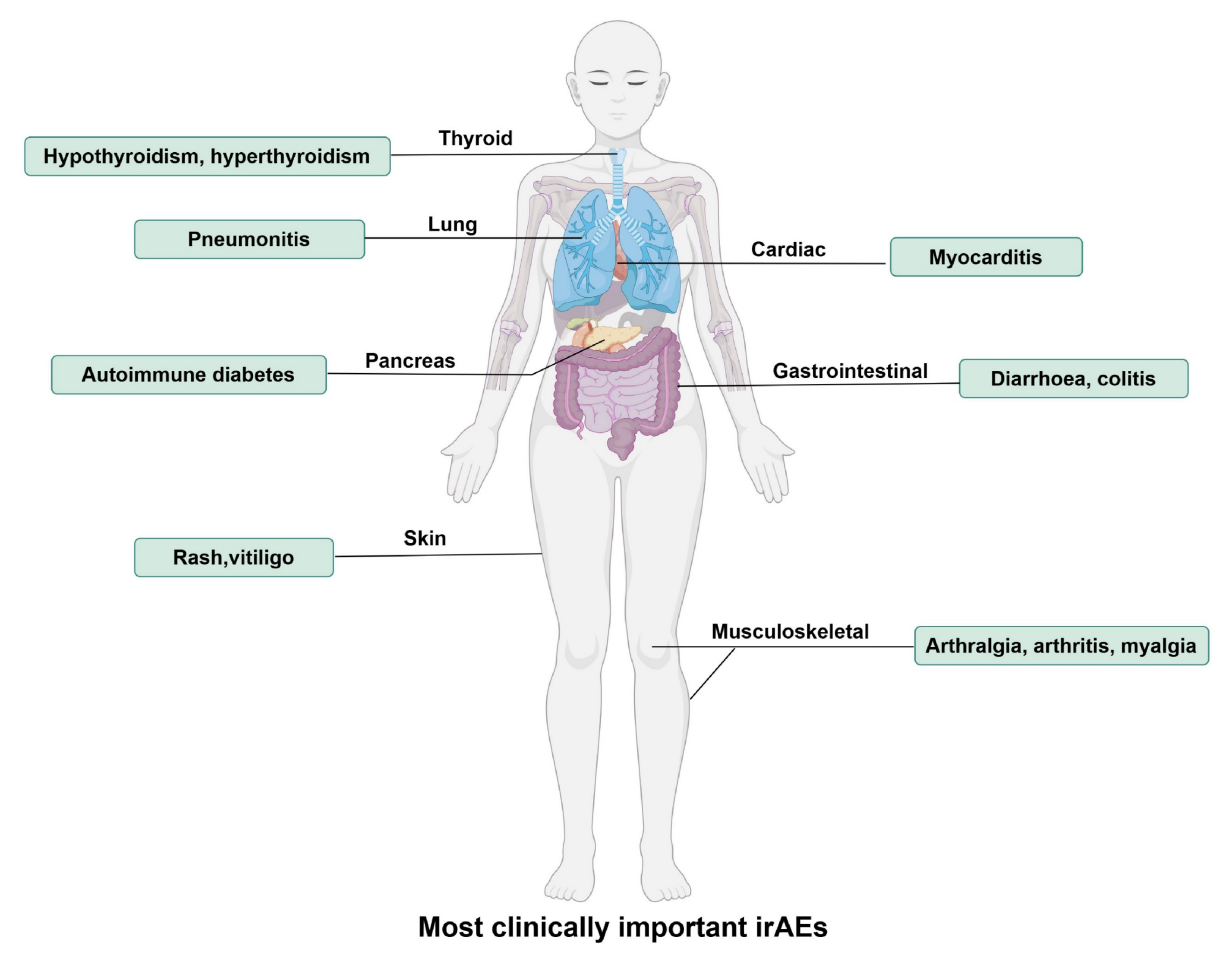
The most clinically important immune-related adverse events (irAEs) for PD-1/PD-L1 blockade therapy. IrAEs affect multiple systems and organs, leading to hypothyroidism, hyperthyroidism, pneumonitis, autoimmune diabetes, rash, vitiligo, myocarditis, diarrhoea, colitis, arthralgia, arthritis, myalgia, etc. (By Figdraw.)

Although PD-1/PD-L1 blockades exhibit anti-tumor effects by activating the immune system, they also lead to an attack of the immune system on normal tissues, and these types of drug-related adverse reactions, mediated by immune mechanisms and which can involve different systems, are referred to as “immune-related adverse events (irAEs)”. The most common irAEs for PD-1/PD-L1 blockade are endocrine (thyroid disorders such as hypothyroidism and hyperthyroidism), gastrointestinal (diarrhea, colitis, nausea), lung (pneumonitis), skin (rash, pruritus, and vitiligo) and musculoskeletal (arthralgia, arthritis, and myalgia), and constitutional symptoms (fatigue, pyrexia, and anorexia) 34, as shown in **Figure 1**. Approximately 20% of patients with PD-1/PD-L1 blockade therapy develop some mild version of gastrointestinal inflammation, with 2-5% developing more severe inflammation 35. Endocrine toxicities are also common with PD-1/PD-L1 blockade therapy. Clinically significant thyroiditis (hypothyroidism) occurs in 8% of patients on PD-1/PD-L1 blockade. Other endocrine toxicities, including autoimmune diabetes and adrenal insufficiency, are rare but are extremely important to recognize because they can be deadly 36. A recent study found that α-myosin was a direct target of cytotoxic CD8^+^ T cells, and α-myosin reactive cells could be expanded from the peripheral blood of patients with immune checkpoint blockade-induced myocarditis 37.

In a meta-analysis regarding PD-1/PD-L1 blockade therapies, among 6,507 patients, 1,111 (17.1%) experienced irAEs of any grade. Among 4,921 patients, 196 (4.0%) experienced irAEs of Grade 3 or higher. Moreover, compared with the use of PD-L1 antibodies, the risk of irAEs occurrence might be higher with the use of PD-1 antibodies 38. Compared to anti-PD-L1, anti-PD-1 therapies are more frequently associated with pneumonitis (2.4% vs 0%), rash (12.2% vs 5.5%), vitiligo (4.0% vs 0%), colitis (0.7% vs 0%), hepatitis (0.4% vs 0%), hypothyroidism (5.1% vs 2.2%), hyperthyroidism (1.6% vs 0%), and anaemia (4.8% vs 0.7%) 34. Current conventional therapy for irAEs includes discontinuation of immune checkpoint blockades and administration of glucocorticoids or infliximab 39.

## Mechanisms of PD-1/PD-L1 Blockade Resistance

Although the PD-1 blockade therapy has shown enormous potential in cancers, in fact, only a small number of patients benefit from its application. For example, when applied to treat advanced recurrent ovarian cancers, PD-1 blockade showed low objective response rates 40. The unsatisfactory response rate limits the application in clinical settings.

The key to the success of PD-1 blocking therapy lies in whether there is resistance to it. Therefore, it is essential to investigate the mechanisms of resistance and low response rates so as to discuss relevant strategies effectively. Based on the clinical response of patients and in order to provide better clinical guidance, the Society for Immunotherapy of Cancer has defined three types of resistance to anti-PD-(L)1 treatment: (1) Primary resistance. It describes disease progression in patients who have been exposed to PD-(L)1 checkpoint inhibitor for at least 6 weeks and have a stable disease period (SD) < 6 months. (2) Secondary resistance. It occurs in patients who have received anti-tumor treatment and have documented, confirmed objective response or extended SD (> 6 months) and then later progress despite continued treatment. (3) The resistance that develops after discontinuation of therapy. It is mainly used to consider other adjuvant therapies after stopping anti-PD-1 treatment and to weigh risk and maximum benefit when deciding whether to cease treatment 41.

**Figure 2:**
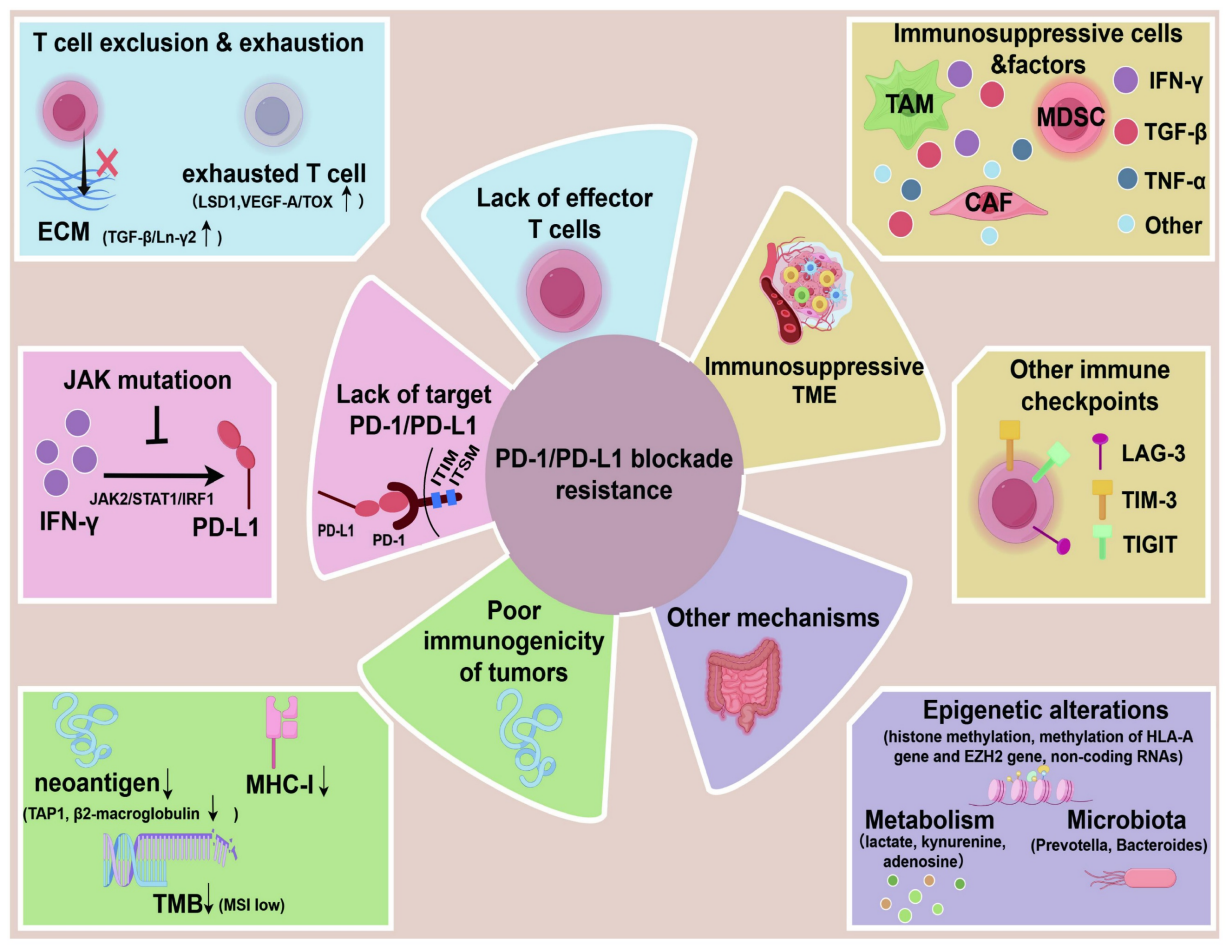
The mechanisms of PD-1/PD-L1 blockade resistance. The main mechanisms include lack of target PD-1/PD-L1(e.g. JAK mutation in tumor inhibit PD-L1 induction by IFN-γ), lack of effector T cells (T cell exclusion and exhaustion), poor immunogenicity of tumors (low neoantigen, low MHC-I, low tumor mutation burden), immunosuppressive TME (immunosuppressive cells, factors, other immune checkpoints), other mechanisms (metabolism, epigenetic alterations, microbitota). (By Figdraw.)

Some scientists have classified the immune resistance mechanisms in the TME into direct, indirect, or other mechanisms 42. Direct resistance is defined as the lack of one or two necessary targets for anti-PD1 therapy: PD-L1 expression and tumor-infiltrating lymphocytes (TILs). For example, compared to type Ⅱ (PD-L1^+^TIL^+^), the types Ⅰ, Ⅲ, and Ⅴ of melanoma may not benefit much from anti-PD1 therapy. It is also referred to as “target-missing” resistance 10. Indirect resistance is non-specific and not unique to anti-PD1 treatments but may be part of the resistance mechanisms in all tumors, such as antigen loss and lack of effective antigen presentation 42. Additionally, there are some other novel mechanisms, such as the gut microbiota, epigenetics, and metabolism. Here, we propose some crucial aspects as below, and also illustrate in **Figure 2**.

### Lack of effector T cells

#### T cell exclusion

Clinically, some “cold” tumors, such as ovarian, prostatic, and pancreatic cancers, show low response to anti-PD-(L)1 therapy. These cold tumors can be immune-desert or immune-excluded. The former lacks T cell infiltration into the tumor and its surroundings, while the latter is defined by T cells being trapped around the tumor periphery or within the stroma, preventing them from sufficient infiltration, which results in a low level of T cells within the tumor 43. Critically, to achieve an effective anti-tumor immune response, there must be sufficient T cell infiltration, making direct physical contact with the tumor 44. Generally, T cell infiltration into the TME correlates with a better prognosis for some cancers. It was found that the 5-year overall survival rate of ovarian cancer patients with T cell infiltration was much higher (38.0%) than those lacking T cell infiltration (4.5%) 45.

Several mechanisms are involved in T cell exclusion, including physical barriers within the tumor stroma, overexpression of TGF-β, and the accumulation of harmful metabolic products within the TME 43. Notably, the stromal cells within the TME contribute to the T cell exclusion, which include cancer-associated fibroblasts (CAFs), myeloid-derived suppressor cells (MDSCs), tumor-associated macrophages (TAMs), and cancer-associated mesenchymal stem cells (CA-MSCs) 44, 46. TGF-β can drive the T cell exclusion within tumors. On the one side, exposure to TGF-β reduces MHC-I expression on cancer cells, while suppression of TGF-β can recover MHC-I expression. On the other side, TGF-β stimulation can activate CAFs, thereby improving the extracellular matrix (ECM), a physical barrier restricting T cell infiltration 47. Moreover, the TGF-β1 signaling derived from CAFs leads to T cell exclusion by upregulating the expression of Ln-γ2, a subunit of laminin, which is a key component of the ECM, thereby constructing a protective barrier for the tumor. This barrier blocks the immune cells' infiltration into the tumor and then reduces the efficacy of anti-PD-1 therapy 48. CA-MSCs can also promote the T cell exclusion. It was confirmed that there was a reverse relationship between CA-MSCs and the CD8^+^ T cell infiltration. CA-MSCs promoted CD8^+^ T cell exclusion within the TME by secreting various chemokines (e.g., CCL2, CX3CL1, and TGF-β1) and restricted T cells within the stroma to reduce the efficacy of PD1 blockade. Under certain situations, CA-MSCs can also differentiate into CAFs 46. Additionally, it was found that the activation of the β-catenin pathway was associated with T cell exclusion in mice with melanoma 49, which was thought to increase PD-1/PD-L1 blockade resistance.

#### T cell exhaustion

T cell exhaustion is a form of dysfunction that T cells gradually acquire when continuously exposed to antigen stimulation. It is a special type of T cells with low reactivity characterized by the loss of effector function (reduced production of IL-2, TNF-α, and IFN-γ, etc.) and the overexpression of various inhibitory receptors on the cell surface (PD-1, CTLA-4, TIM-3, etc.). Blocking these inhibitory receptors can partially reverse T cell exhaustion 50. However, PD-1 blocking therapy can also induce T cell exhaustion, which may be one of the reasons for resistance. It was found that anti-PD-1 therapy promoted the progenitor exhausted CD8^+^ T cells' proliferation and differentiation *in vivo*, ultimately leading to an increase in terminally exhausted cells but short lifespans 51. Additionally, it was revealed that increased collagen levels in lung tumors were involved in PD-(L)1 blockade resistance. This process was associated with the leukocyte-associated immunoglobulin (Ig)-like receptor (LAIR1), inhibiting lymphocytes through SHP-1 signaling. The reduction of tumor collagen deposition increased T cell infiltration, diminished T cell exhaustion, and eliminated resistance to anti-PD-L1 therapy 52. Another study discovered immunosuppressive CD10^+^ALPL^+^ neutrophils mediated resistance to anti-PD-1 immunotherapy by inducing an irreversible T cell exhaustion 53. The lysine-specific demethylase 1 (LSD1) could inhibit the progenitor pool and promote T cell exhaustion within the TME by antagonizing TCF1-mediated transcription. Inhibition of LSD1 could enhance the progenitor phenotype, thereby promoting a sustained response to anti-PD-1 and avoiding resistance 54. In addition, researchers found that in microsatellite stable colorectal cancers that were lowly responsive to PD-1 blockade, VEGF-A induced the expression of transcription factor TOX-mediated T cell exhaustion. T cell exhaustion could be recovered after knocking down TOX, and the combination with anti-VEGF-A treatment could improve the response to PD-1 blockade therapy 55.

### Lack of target PD-1/PD-L1

During tumor progression, once tumor cells express tumor antigens that can be recognized by immune cells, especially T cells, they will face the immune attack. To escape this immune attack, PD-1 and PD-L1 are upregulated under TME stimulation, which contribute to the resistance to immune response and immune surveillance evasion. Currently, the prevailing view is that PD-1 protein is rapidly expressed on activated effector T cells by TCR stimulation and is also regulated by various factors and pathways like TGF-β, IL-12, IL-6, IFN-α, TNF-α, etc^56^. PD-L1 protein is seldom constitutively expressed in normal tissues and cultured tumor cell lines, but it can be found in the majority of cancer specimens, suggesting a latent role of the TME in regulating PD-L1 expression 22. Specifically, PD-L1 is selectively upregulated within the TME by IFN-γ released from T cells, which may be due to various mechanisms, such as IFN-γ leads to the activation of JAK2/STAT1/IRF1, which subsequently upregulates PD-L1 expression 21. However, the signaling pathways through which IFN-γ induces PD-L1 differ among various tumor types 56.

Mechanistically, PD-1 and PD-L1 blockades exert anti-tumor effects by inhibiting the negative regulatory effects on T cells via binding with PD-1 and PD-L1, respectively. Therefore, the expression of PD-1 or PD-L1 is indispensable for this therapy. A study indicated that melanoma patients with high tumor burden and JAK1/2 mutations are insensitive to PD-1/PD-L1 blockade, partly because the JAK1/2 mutation hindered the adaptive upregulation of PD-L1 when exposed to IFN-γ^57^. Consistently, patients with melanoma who are both PD-L1 positive and TILs positive are the most effective to PD-1 blockade therapy 58. Currently, it is imperative to identify specific patient types before initiating anti-PD-(L)1 therapy to avoid ineffective treatment. For instance, patients with PD-L1 negative may need to consider other immunotherapies in addition to PD-1/PD-L1 blockade 10. Although PD-L1 positive is suggestive for therapeutic efficacy, it is still worth noticing that the expression levels of PD-L1 are incorrectly measured sometimes due to tumor heterogeneity, dynamic changes of PD-L1, lack of validated biomarkers, and inaccurate biopsy sampling 8, 59.

Although PD-L1 positive expression is considered as a marker of better response to anti-PD-(L)1 therapy, PD-L1 in exosomes may contribute to resistance to anti-PD-(L)1 therapy. Higher levels of circulating exosomal PD-L1 before treatment are negatively correlated with the response, indicating T cell exhaustion at a stage that is irreversible by anti-PD-(L)1 therapy. Thus, the levels of exosomal PD-L1 can distinguish patients who will respond to anti-PD-(L)1 treatment 60. It was found that either the exosome inhibitor GW4869 or the suppression of Rab27a to reduce exosome release improved the efficacy of anti-PD-L1 treatment 61. This might be due to the fact that exosomal PD-L1 depleted antibodies, prevented the availability of surplus antibodies to inhibit PD-L1, which eventually affected the efficacy of anti-PD-L1 therapy 62.

### Poor immunogenicity of tumors

It is well known that antigens that are normally presented do not lead to any immune attack. However, when anomalies such as DNA mutations happen and these antigens are presented on the cell surface, immune cells could identify the tumor cells, eliciting the anti-tumor response. The tumor immunogenicity influences the identification of T cells. Therefore, the tumor immunogenicity is crucial to the efficacy of PD-1/PD-L1 blockade.

#### Loss of neoantigens and antigen presentation

“Tumor immunoediting” includes three stages: immune elimination, immune equilibrium, and immune escape. At the immune elimination stage, T cells eliminate tumor cells that strongly express antigens. In contrast, at the immune evasion stage, in the face of intense selective pressure by the immune system, tumors may evade attack from the immune system by losing the expression of antigens 63. To trigger an effective anti-tumor immune response, two crucial steps are necessary. Firstly, tumor cells process and present tumor antigens via MHC-I. Secondly, APCs take up the antigen and mediate cross-presentation to activate CD8^+^ T cells 64.

Neoantigens, as a type of tumor-specific antigen, can enhance the sensitivity to immune checkpoint blockade therapy, while the deficiency often leads to immune escape. Cancers with abundant mutated neoantigens show increased sensitivity to PD-1 inhibitors 65. Many mechanisms can influence antigen presentation, such as regulating the function of dendritic cells, HLA expression levels, and other genes' expressions that are involved in antigen presentation. It was found that downregulated MHC-I may be a marker of resistance to PD-1 blockade 66. Furthermore, there is an opinion that a dysfunction within the components of the Antigen Processing Machinery (APM), such as low expression or silencing of TAP1, β2-microglobulin, or HLA-A, HLA-B, and HLA-C, may hinder the presentation of neoantigens on the cell surface and also affect the infiltration of CD8^+^ cells 67. A study found that Nintedanib enhanced the efficacy of PD-L1 blockade therapy by increasing the levels of PD-L1 and MHC-1 expressed on tumor cells 68. Another research discovered that the EZH2 inhibition enhanced the presentation of antigens by upregulating MHC-1 expression so as to increase the sensitivity to PD-1 blockade therapy 69.

#### Low tumor mutation burden

There is an apparent correlation between the tumor mutation burden (TMB) and the objective response rate of PD-1/PD-L1 blockade. TMB may be one of the reasons for the different outcomes in different cancers 70. Generally, errors in DNA replication can be corrected in normal cells through a mechanism called mismatch repair (MMR). However, the absence of MMR can lead to easier accumulation of mutations, thereby increasing tumor immunogenicity. Microsatellite instability (MSI) is one of the important indicators of MMR deficiency 71. Clinically, in patients with metastatic MSI-High-dMMR colorectal cancer, pembrolizumab (anti-PD-1) treatment extended the duration of progression-free survival 6. A multi-center study on advanced NSCLC patients revealed that elevated TMB increased the response to PD-1/PD-L1 blockade and improved overall survival in those with high PD-L1 expression 72. Similarly, in patients with advanced solid tumors, it revealed that objective response to pembrolizumab was observed in 29% (30 out of 102) with high TMB and in 6% (43 out of 688) without high TMB, suggesting that high TMB levels may be applied to distinguish patients who would benefit significantly from anti-PD-1 treatment 73. In addition, TMB was found to be significantly higher in melanoma patients who responded to combination blockades with anti-CTLA-4 and anti-PD-1 than in those who did not respond 74. All of the above suggest that the elevated TMB is positively correlated with the efficacy of anti-PD-1, and patients with high TMB show high sensitivity, while those with a low TMB often show low sensitivity.

### Immunosuppressive TME

To resist the anti-tumor immune response, the tumor educates an immunosuppressive TME, whose components can affect anti-tumor immunotherapy, such as immunosuppressive cells, cytokines, and some co-inhibitory receptors. The immunosuppressive TME partly accounts for the resistance to PD-1/PD-L1 blockade therapy 42.

#### Immunosuppressive cells

Currently, it is known that there are some immunosuppressive cells in the TME, such as TAMs, CAFs, and MDSCs. TAMs exist as M1 and M2 types, usually the M2 type, which is often associated with tumor progression and poor outcomes 75. TAMs can influence immunotherapy and contribute to immune resistance through various mechanisms 76. (1) Upregulation of immune checkpoints, primarily PD-L1. One study found that M2-type TAMs promoted PD-L1 overexpression in gastric cancer cells via M2-Exos exosomes, which eventually promoted the growth and invasion of cancer cells 77. Another study found that TAMs were the primary source of PD-L1 in the murine cholangiocarcinoma model, with approximately 60% of TAMs expressing PD-L1^78^. High expression of PD-L1 in TAMs indicated an activated immune microenvironment, often accompanied by abundant CD8^+^ T cell infiltration as well as high expression of immune-related genes 79. In addition, TAMs also expressed other immune checkpoints that collaborate with PD-L1 to promote immune evasion, such as VISTA 75. (2) Crosstalk between regulatory T cells (Tregs) and TAMs: On the one hand, TAMs recruit Tregs into the TME through some chemokines (such as CCL20), cytokines, and exosomes. On the other hand, Tregs can promote the immunosuppressive function of TAMs 76. (3) Hijacking anti-PD-1 antibody: TAM can capture anti-PD-1 antibody through binding to the Fc region of the antibody with the FcγR expressed on the macrophages 80. (4) Influencing T cell activation and function. TAMs express the transcription factor IRF8, which is necessary for antigen presentation by TAMs, and can lead to the depletion of cytotoxic T lymphocytes (CTLs) within the TME. The specific absence of IRF8 in TAMs can prevent the exhaustion of cancer-cell-reactive CTLs and inhibit tumor growth 81. (5) Secreting regulatory cytokines. TAMs can promote immune suppression and affect the PD-(L)1 blockade efficacy by secreting regulatory cytokines (such as TGF-β, PGE2, IL-6) 75.

CAFs can affect PD-1/PD-L1 immunotherapy by secreting various cytokines (such as WNT2, TGF-β1, CXCL5) and extracellular vesicles. They also regulate other immune cells within the TME and participate in the ECM remodeling, eventually leading to the failure of anti-PD-(L)1 therapy 82. MDSCs, as a group of immature myeloid cells, are considered to be negatively correlated with the efficacy of immune checkpoint blockade due to their ability to suppress T cell activity and express PD-L1. Targeting MDSCs could boost the efficacy of anti-PD-1 therapy 78.

#### Immunosuppressive factors

Within the TME, there are cytokines that can negatively regulate the efficacy of anti-PD-(L)1 therapy. For instance, cytokines such as IFN-γ, TGF-β, TNF-α, ECF and GM-CSF can promote tumor immune evasion by regulating the expression of PD-L1^56^.

IFN-γ is essential in regulating PD-L1 expression, commonly secreted by CD4^+^ T cells differentiated into Th1. It plays a vital role in innate and adaptive immune responses against pathogens and tumors 83. To exhibit its function, IFN-γ must bind to its receptor IFNGR (includes IFNGR-1 and IFNGR-2), and the activation of IFN-γ signaling is mainly mediated through the JAK-STAT pathway 83. IFN-γ is essential for “tumor immunoediting” 83, 84. At the stage of immune elimination, IFN-γ acts as an immune-stimulating molecule, together with other components in the TME, promoting the immune system to recognize and eliminate the tumor cells that strongly express antigens. For instance, IFN can induce the conversion of TAMs from pro-tumor M2 type to the anti-tumor M1 type 85. At the stage of immune equilibrium, IFN-γ is essential for maintaining the dormancy and balance of tumors. At the stage of immune evasion, IFN-γ acts as an immunosuppressive molecule, promoting tumor cells to evade the immune system surveillance. Besides PD-L1, IFN-γ also promotes the expression of other immune checkpoints on T cells, such as LAG-3 and CTLA-4^83^. The modulation of tumor immunity by the interferon signaling pathway is complex. It was revealed that when exposed to IFN-γ or IFN-β, B16 tumor cells with an IFN-γ deficiency showed a more significant growth tendency compared to wild-type tumor cells. Ptpn2 negatively regulated IFN-γ signaling by dephosphorylating JAK1 and STAT1. That loss of Ptpn2 enhanced IFN-γ signaling and antigen presentation to T cells, leading to increased sensitivity to immunotherapy 86. Another study found that the deletion of JAK2 led to a decrease of tumor suppressor gene CDKN2A expression, which increased the susceptibility of tumors to develop resistance to IFN-γ and immunotherapy 87.

Similarly, TGF-β can shape an immunosuppressive TME resistant to anti-PD-(L)1 therapy by regulating the activity of various immune cells, promoting epithelial-mesenchymal transition, and promoting T cell exclusion 88. That dual inhibition of TGF-β and PD-1 could increase the anti-tumor efficacy. Moreover, some chemokine receptors, such as CCR1, CCR2, CCR4, and CCR5, have been proven to be associated with tumor immunosuppression. Combined inhibition of these chemokines with anti-PD-(L)1 treatment can improve the therapeutic efficacy 89.

#### Other immune checkpoints

Within the TME, other immune checkpoints, such as TIM-3, LAG-3, and TIGIT, are also considered to be related to anti-PD-(L)1 resistance. Specifically, one study indicated that PD-1 blockade upregulated other immune checkpoints, such as LAG-3 and CTLA-4, which may affect the efficacy of monotherapy 90. However, this compensatory upregulation is likely to be overcome by combination blocking strategies, and even to some extent, the upregulated immune checkpoints may provide more targets for antibody blockade. Therefore, combination therapy may exhibit significant synergistic or combined effects. In mouse model of NSCLC, the upregulation of TIM-3 was found to be related to anti-PD-1 resistance, and blocking TIM-3 could boost the efficacy of anti-PD-1 therapy 91. The following section will discuss the application of other ICBs in combination with PD-1/PD-L1 blockade in cancer therapy.

### Other mechanisms

#### Metabolism

The rapid generation of ATP meets the high metabolic growth demands of the tumor, while the substantial accumulation of lactic acid helps to construct an immunosuppressive TME 92. For instance, studies showed that lactate induced the conversion of macrophages into M2 type and promoted lung cancer progression, and the accumulation of lactic acid prevented T cell infiltration into the TME and reduced IFN-γ expression 93. It is also worth noting that the high consumption of glucose in tumor cells may restrict the glycolytic activity in T cells, leading to T cell dysfunction. In addition, tumor cells also promote immune suppression by secreting some inhibitory metabolic products, such as kynurenic acid, adenosine, and PGE2^92^. Collectively, all of these factors are conducive to tumor immune evasion and are related to anti-PD-(L)1 resistance.

#### Epigenetic alterations

Epigenetic changes refer to the regulation of gene expression without altering the DNA sequence, mainly involving DNA methylation, histone modification, RNA modification, and chromatin remodeling. These epigenetic changes can be involved in anti-tumor immunity 94. For example, it was found that the downregulation of MHC-I in ovarian cancer cells may be related to epigenetic mechanisms. Specifically, there was a strong inverse correlation between the expression of the HLA-A gene and the level of promoter methylation 47. Another study found that histone methylation and DNA methylation of EZH2 suppressed the production of Th1 chemokines CXCL9 and CXCL10, and reduced effector T cell infiltration into the TME in mice with ovarian cancer. Consequently, inhibition of this epigenetic process was believed to boost anti-PD-L1 efficacy 95. In recent years, some non-coding RNAs exhibited potential effects on the efficacy of PD-1/PD-L1 blockade therapy 96. For instance, circHMGB2 could remodel the TME by upregulating the expression of CARM1 by sponging miR-181a-5p and limiting anti-PD-1 efficacy in NSCLC. Inhibition of CARM1 improved the sensitivities of NSCLC cells with high circHMGB2 expression to anti-PD-1 treatment 97.

#### Microbiota

Gut microbiome is thought to influence the efficacy of PD-1/PD-L1 blockade therapy 98. For example, an analysis of the gut microbiome in patients with gastrointestinal tumors treated with anti-PD-1/PD-L1 showed that responders had an increased ratio of the relative abundance of Prevotella and Bacteroides, and the relative abundant types of bacteria varied with different tumors 99. PD-L1 blockade was significantly effective in melanoma mice with microbiome transplantation from responders to anti-PD-1 therapy, but was completely ineffective in mice with microbiome transplantation from non-responders 98. Additionally, it was revealed that antibiotics suppressed the efficacy of anti-PD-1 therapy in patients with advanced cancer. Notably, fecal microbiota transplantation (FMT) from patients who responded to anti-PD-1 therapy into antibiotic-treated mice improved the efficacy of anti-PD-1, while FMT from non-responders did not improve the efficacy 100.

## PD-1/PD-L1 Blockade in Combination with Other ICBs

**Figure 3:**
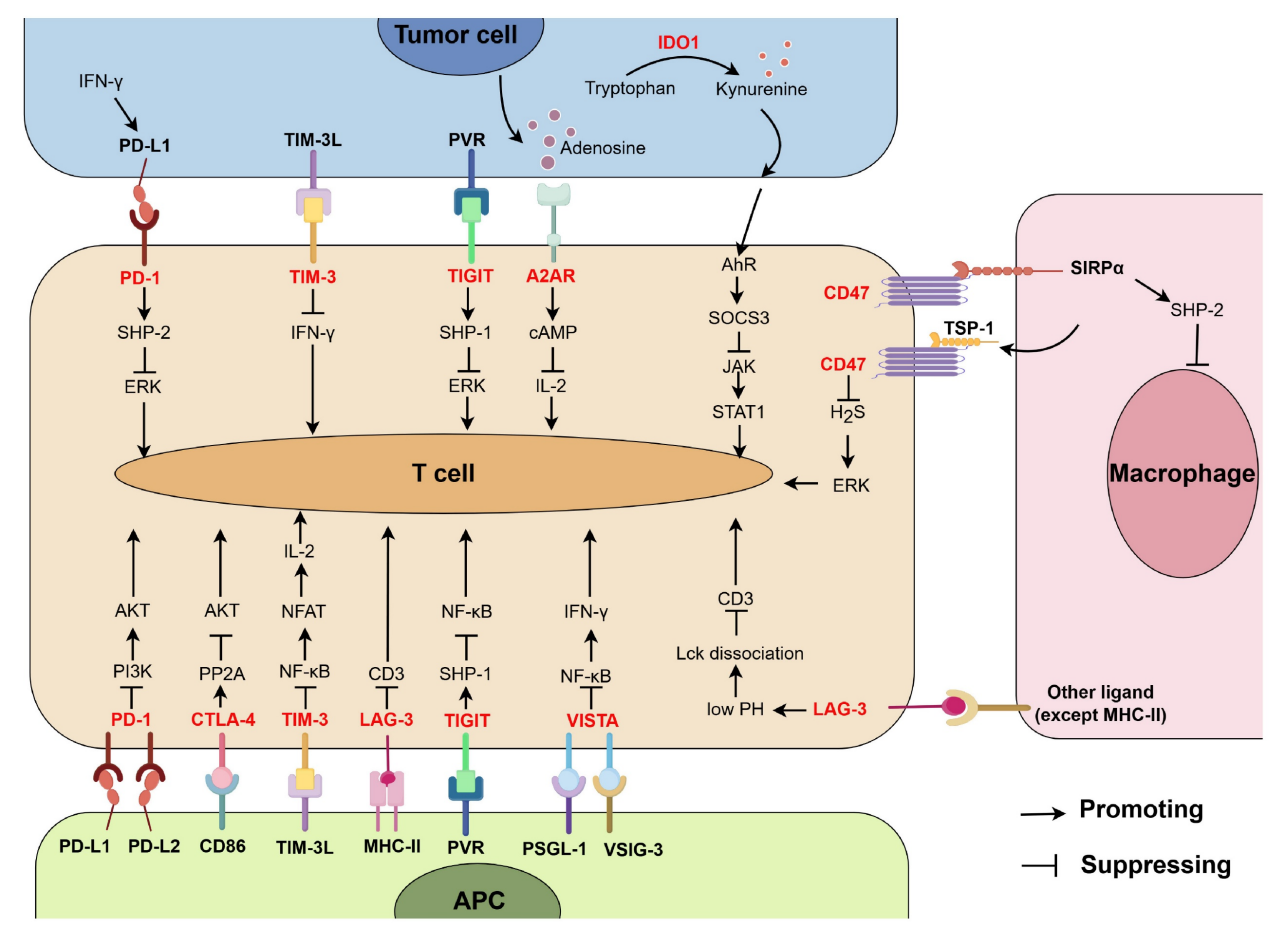
The distribution T cell-related immune checkpoints (highlighted in red) and their function. Receptors PD-1, CTLA-4, TIM-3, LAG-3, TIGIT, VISTA, A2AR, and CD47 interact with their ligands, then lead to suppression of T cell activation through different pathways. IDO1 promotes the production of kynurenine, which in turn binds with receptor AhR and suppresses T cell activation via SOCS3/JAK/STAT1 axis. (By Figdraw.)

Combined application of PD-1/PD-L1 blockade with other treatments offers potential promising prospects for improving the low response rate of PD-1/PD-L1 blocking therapy and overcoming the drug resistance. Below, we will focus on the synergistic anti-tumor response of the combined blockades of PD-1/PD-L1 and other immune checkpoints, and the distribution of these immune checkpoints and their function are summarized in **Figure 3**.

### CTLA-4

CTLA-4 (cytotoxic T-lymphocyte associated antigen-4, CD152), a receptor that is upregulated on activated T cells. CTLA-4 has a high resemblance to CD28 and binds to B7-1 (CD80) and B7-2 (CD86) on APCs with a higher affinity than CD28. As a co-inhibitory receptor, it competes with CD28, negatively regulating T cell activation 101. Furthermore, CTLA-4 is mainly constitutively expressed on Tregs or induced after T cell activation in response to CD28 and TCR signaling 102. Additionally, CTLA-4 induces the expression of indoleamine 2,3-dioxygenase 1 (IDO1) by activating the noncanonical NF-κB pathway, then the expression of IDO further promotes the differentiation of Tregs, which could inhibit the activation of other T cells, thereby forming an immunoregulatory network 103.

The blockade of CTLA-4's suppressive function allows and enhances the effective immune response against tumor cells 104. In 2011, the first monoclonal antibody against CTLA-4 ipilimumab received FDA approval. Anti-CTLA-4 may exert its anti-tumor effects by increasing the activity of effector CD4^+^ T cells and inhibiting the Tregs-dependent immune suppression 32. It was found that anti-CTLA-4 treatment mainly affected CD4^+^ T cells 32, and induced cytotoxic CD4^+^ T cells in melanoma patients 105. Moreover, Tregs, a subset of CD4^+^ T cells, are the primary targets of anti-CTLA-4 for anti-tumor efficacy. High frequencies of Tregs before anti-CTLA-4 treatment are correlated with better response to anti-CTLA-4 therapy 106. Additionally, the reduction in FoxP3/Tregs levels during treatment with ipilimumab treatment was linked to a better clinical outcome and survival rate 107.

The advantage of the combination of anti-CTLA-4 and anti-PD(L)1 therapy is that they affect T cell activation through different mechanisms, which may complement each other in mechanism and efficacy. (1) Affecting different subsets of TILs. Anti-PD-1 predominantly leads to the proliferation of exhausted-like CD8^+^ T cells within the tumor, whereas CTLA-4 inhibitor mainly results in the amplification of CD4^+^ effector cells and exhausted-like CD8^+^ T cells 19. It was found that the combination of anti-PD-L1 and anti-CTLA-4 treatment had a stronger inhibitory effect in colon cancer than monotherapy. Both the anti-CTLA-4 monotherapy and the combined treatment increased CD4^+^ and CD8^+^ T cells significantly in the tumors, and decreased intra-tumoral Tregs 108. (2) Affecting the activation of T cells at different stages. CTLA-4 is rarely expressed on naïve T cells, but is upregulated on activated T cells and reaches a peak within 48-72 hours. Thus, CTLA-4 may play a role after the initial T cell activation 109. In addition, in another study, the expression levels of CTLA-4 in active T cells peaked on the third day and returned to baseline levels by the seventh day, while the PD-1 expression gradually increased over time, reaching the maximum level on the tenth day 110. (3) Affecting T cell activation in different regions. PD-1 is highly expressed on activated T cells and requires binding to its ligand PD-L1 to exert its effects, which mainly restricts T cell activity at specific tissue sites, such as the TME. In contrast, CTLA-4's binding is not dependent on ligands, primarily competing with CD28 for the binding of B7-1 and B7-2. A study found that CTLA-4 on Tregs interacted with CD80 expressed on dendritic cells within the lymph nodes surrounding the tumor to regulate CD4^+^ T cell infiltration into tumors. Treatment with anti-CTLA-4 stimulated CD4^+^ T infiltration into the tumor, suggesting the inhibitory effect of CTLA-4 on T cells in lymph nodes 111. Similarly, another study demonstrated that the expansion of TCR in melanoma patients from CTLA-4 blockade (tremelimumab) is related to the priming encounter between T cells and APCs in the lymph nodes, whereas the inhibitory signal of PD-1/PD-L1 with PD-1 blockade (pembrolizumab) occurs in peripheral tissues 112. (4) Inhibiting the PI3K/Akt pathway by different ways. PD-1 blocks the induction of PI3K activity, which is essential for the activation of Akt, and this inhibition by PD-1 is dependent on its ITSM motif. In contrast, CTLA-4 directly inhibits Akt through the activation of phosphatase PP2A, while retaining the activity of PI3K 113. These findings indicate that the two blockades may have synergistic or additive effects in inhibiting the PI3K/Akt pathway. (5) Overcoming the upregulation of immune checkpoints induced by monotherapy. For instance, a study showed that the CTLA-4 expression in CD4^+^ Tregs increased after the administration of the blocking antibody, and PD-1 expression in CD8^+^ T cells also increased, especially in the group treated with one blockade 114. (6) Remodeling a more advantageous tumor immune microenvironment. It was revealed that the dual blockades of PD-1 and CTLA-4, in contrast to individual inhibition, significantly enhanced the CD8^+^ T cells/Tregs and CD8^+^ T cells/MDSCs ratios within the tumor in mice with melanoma. The increased effector T-cell (Teff) within the tumor seemed to be associated with high levels of inflammatory cytokine levels such as IFN-γ and TNF-α in the relevant tissues induced by the dual blockade 114. Furthermore, in another study, it was discovered that dual blockades of PD-1 and CTLA-4 increased IFN-γ production, reduced the secretion of immunomodulatory cytokines such as TGF-β and IL-10, promoted TIL proliferation and its cytolytic function through upregulation of ribosomal S6 kinase (S6K), and two T-box transcription factors T-bet, and Eomes which regulated Th1 and cytolytic function of CD8^+^ cells 115.

To sum up, CTLA-4 expression in T cells is increased after anti-PD-(L)1 treatment. The combination of anti-CTLA-4 and anti-PD-(L)1 therapy is because that they affect T cell activation through different mechanisms, affecting different subsets of TILs at different stages and in different regions, so that they play more effective anti-tumor effects.

### TIM-3

T cell immunoglobulin-3 (TIM-3, CD366, HAVCR2), serving as a co-inhibitory receptor, is involved in the immune regulation of autoimmune diseases, transplantation tolerance, tumors, and infectious diseases 116. Initially, it was identified to negatively regulate the generation of Th1 and Tc1 cells that secrete IFN-γ, reducing their apoptosis. Subsequently, the research revealed its expression on innate immune cells, including dendritic cells, macrophages, and NK cells 117. It has been confirmed that the ligands for TIM-3 include Galectin-9, PtdSer, CEACAM1, and HMGB1. Unlike classic immune checkpoints such as PD-1, TIM-3 does not have the inhibitory signaling motifs in its cytoplasmic tail, such as ITIMs or ITSMs, but it contains five conserved tyrosine residues 118. TIM-3 could inhibit TCR-mediated signaling by suppressing the NF-κB/NFAT signaling pathway in Jurkat T cells and primary human CD8^+^ T cells, thereby inhibiting IL-2 secretion and T cell activation 119. The high expression level of TIM-3 was found to be associated with T cell exhaustion and dysfunction within tumors 120.

Interestingly, TIM-3 was found to be co-expressed with PD-1, and the combined blocking of PD-1 and TIM-3 had a collaborative effect on enhancing effector T cells' function and their ability to destroy tumor cells 118. The combined blocking increased the frequency of IFN-γ, TNF, IL-2-producing NY-ESO-1-specific CD8^+^ T cells and the frequency of proliferating and total NY-ESO-1-specific CD8^+^ T cells, suggesting a collaborative effect between TIM-3 and PD-1 blockade 120. The co-expression of TIM-3 and PD-1 was found to be associated with poor prognosis of colorectal cancer 121 and gallbladder cancer 122. Additionally, in mouse model of NSCLC, it was revealed that the upregulation of TIM-3 might be one of the mechanisms of anti-PD-1 resistance. Treatment with TIM-3 blockade after the failure of PD-1 blockade therapy can significantly enhance the anti-tumor efficacy and survival benefit. Furthermore, Galectin 9 (one of the ligands of TIM-3) was found to be significantly elevated in PD-1-resistant tumor samples at both RNA and protein levels. In addition, the combination therapy increased IFN-γ production and proliferation of TIM-3^+^CD8^+^ T cells from PD-1-resistant mice, as well as decreased the expression of some tumor-promoting cytokines, such as IL-6 and progranulin 91. Studies showed that dual blocking of PD-1 and TIM-3 simultaneously exerted better anti-tumor effects compared to blocking either one alone. In a study about mice with acute myeloid leukemia, it was discovered that the combination of TIM-3-Fc fusion protein and anti-PD-L1 significantly reduced tumor burden at all time points and extended the survival of wild-type mice with high tumor load, compared to single blockade treatment 123.

In addition, it was found that the expression of PD-1 and TIM-3 was significantly upregulated in CD4^+^ and CD8^+^ T cells isolated from tumor tissues and ascites of patients with hepatocellular carcinoma. The combined blocking exhibited a more significant anti-tumor effect than a single antibody blockade in mice with hepatocellular carcinoma through increased production of T cell effector cytokines (IFN-γ and TNF-α) and TILs, decreased levels of immunosuppressive cytokines (IL-10 and IL-6), and the amounts of PD-1^+^TIM-3^+^CD8^+^ T cells within the TME, which are related to tumor immune evasion 124. Thus, dual blocking of PD-1 and TIM-3 exerts better anti-tumor effects compared to blocking one alone, and the co-expressed TIM-3 with PD-1 is the possible mechanism.

### LAG-3

The lymphocyte activation gene 3 (LAG-3), also known as CD223, is frequently expressed on various activated immune cells, including CD4^+^ and CD8^+^ T cells, Tregs, NK cells, B cells, and dendritic cells. It binds with several classical ligands, such as MHC-II, Galectin-3, LSECtin, alpha-synuclein, and FGL1. The engagement with these ligands can induce a state of exhaustion in the respective immune cells 125. Specifically, LAG3 binds to MHC-II and inhibits T cell proliferation and cytokine production through the association with CD3 in the TCR-CD3 complex 126. Moreover, LAG3 may serve as a signal disruptor in the absence of its canonical ligand MHC-II. Mechanistically, a tandem glutamic acid-proline repeat in the LAG3 cytoplasmic tail lowered the pH at the immune synapse and caused dissociation of the tyrosine kinase Lck from the CD4 or CD8 co-receptor, which resulted in a loss of co-receptor-TCR signaling and limited T cell activation 127. Therefore, the blockade of LAG-3 has shown promising therapeutic effects in various types of malignant tumors, and anti-LAG-3 immunotherapeutic agents have been utilized to restore T cell function.

Notably, the combination of LAG-3 with other ICBs may yield better outcomes. In cancers, T cells are persistently stimulated by antigens, leading to the continuous high expression of LAG3 along with other co-inhibitory receptors such as PD-1, CTLA-4, and TIM-3. This results in T cell exhaustion, which is characterized by decreased cytokine production, reduced proliferative capacity, and diminished ability to kill tumor cells 128. In 2022, the FDA approved Opdualag (a fixed-dose combination of LAG-3 blocking antibody relatlimab and PD-1 blocking antibody nivolumab) for the treatment of unresectable or metastatic melanoma 129. One study showed that LAG3 and PD-1 were upregulated and co-expressed in TILs from mice bearing ovarian tumors. Dual blockade or gene knockout of LAG-3 and PD-1 produced high levels of IL-2, IFN-γ, TNF-α, and granzyme B, increased the percentage of CD8^+^ and CD4^+^ TILs, enhanced the effector function of CD8^+^ T cells, reduced the frequency of suppressive Tregs within the TME so that it delayed tumor growth and extended the life span of mice significantly 130. Similarly, another study found that the expression of multiple immune checkpoints was upregulated in tumor-associated lymphocytes (TALs) isolated from patients with ovarian cancer, and the co-expression of PD-1 and LAG-3 was the most significant in CD8^+^ TALs, similar to the findings observed in mouse model 90. Furthermore, in patients with advanced NSCLC, overexpression of LAG-3 was found to be negatively correlated with the survival benefit from PD-1/PD-L1 blockade 131. To sum up, the combination of LAG-3 blockade with PD-1/PD-L1 blockade exhibits better anti-tumor effects than PD-1/PD-L1 blockade alone in clinic and preclinical studies, and this might be due to the co-expression of LAG3 and PD-1 at high levels in TILs within the TME.

### TIGIT

T cell immunoreceptor with immunoglobulin and ITIM domain (TIGIT), also known as Vstm3 or VSIG9, is an immune checkpoint receptor expressed on T cells and NK cells. Its structure includes an immunoglobulin variable domain, a transmembrane domain, and an ITIM 132. The ligands identified for TIGIT include poliovirus receptor (PVR, CD155), PVRL2 (CD112), and PVRL3 (CD113), and PVR shows the highest affinity to TIGIT. Studies have demonstrated that TIGIT effectively competes with both CD226 (an activating receptor) and CD96 (an inhibitory receptor) for binding to their shared ligand PVR.^132^. TIGIT has a variety of actions: (1) After binding to PVR, TIGIT recruits SHIP-1 to inhibit the activation of the NF-κB and ERK signaling pathways, thereby reducing cytokine production and leading to the exhaustion of CD8^+^ T cells 133. (2) The interaction between TIGIT and PVR induces IL-10 production in dendritic cells, thereby suppressing T cell activation 132. (3) TIGIT competes with CD226 to bind PVR, thus inhibiting CD226-mediated T cell activation. (4) The expression of TIGIT in Tregs enhances their immunosuppressive function and stability 134. (5) Tumors exploit the Fap2 protein of Fusobacterium nucleatum to interact with TIGIT, inhibiting the activation of T cells and NK cells 135.

Recent studies found that the combined targeting TIGIT and PD-1 outperformed single blocking in terms of tumor suppression. For example, one study found that TIGIT and PD-1 co-expressed on most NY-ESO-1-specific CD8^+^ T cells isolated from melanoma patients, and dual blockades of TIGIT and PD-1 further increased NY-ESO-1-specific CD8^+^ T cell counts. Additionally, it found that PD-1 blockade increased TIGIT expression, but TIGIT blockade could not increase PD-1 expression 136. Moreover, another study found that in the surgical resection samples of glioblastoma, the TILs exhibited similar expression levels of PD-1 and TIGIT. Additionally, in mice with implanted tumors, it was observed that the long-term survival rate for the group treated with single PD-1 blockade was 16.7%, while the control group and the single TIGIT blockade group had a long-term survival rate of 0%. Surprisingly, the dual blockade of both targets increased the long-term survival rate to 48.0%, providing a greater survival benefit. The combined blockade and PD-1 single blockade could establish anti-tumor immune memory, and the combination blockade therapy provided more significant efficacy, which may be related to increased infiltration of CD8^+^ and CD4^+^ T cells, increased production of cytokines IFN-γ and TNF-α, and decreased tumor-infiltrating dendritic cells 137. In a word, the dual blockade of TIGIT and PD-1 provides a greater survival benefit than PD-1/PD-L1 blockade alone. This might be due to the fact that PD-1 blockade increases TIGIT expression.

### VISTA

V-domain Ig suppressor of T cell activation (VISTA), a transmembrane protein, is also known as PD-1 homologue (PD1H) due to the high homology of its IgV domain with the CD28 and B7 families 125. There are several identified ligands for VISTA, such as PSGL-1, VSIG-3, Galectin-9, LRIG1, and Syndecan-2^138^. VISTA has been discovered with high expression levels in tumor-infiltrating myeloid cells, including myeloid dendritic cells and MDSCs. Moreover, blockade of VISTA was also found to impair the inhibitory effect of Tregs, but it was not clear whether this was related to the expression of VISTA on Tregs. Additionally, VISTA was constitutively expressed on naïve T cells, maintaining their resting state and inhibiting T cell activation 139. After binding to PSGL-1, VISTA was able to inhibit the phosphorylation of NF-κB in T cells, thereby reducing the production of cytokines (such as IFN-γ) and suppressing the proliferation and activation of T cells 140. VSIG-3 also acted as a ligand for VISTA. The interaction between VSIG-3 and VISTA significantly reduced the production of cytokines and chemokines by T cells, including IFN-γ, IL-2, IL-17, CCL5/Rantes, CCL3/MIP-1α, and CXCL11/I-TAC, and also inhibited the proliferation of T cells activated by anti-CD3 antibodies 141.

Interestingly, VISTA blockade was found to exhibit a cooperative effect with anti-PD-(L)1 therapy in multiple cancer types. For example, the combined administration of anti-VISTA and anti-PD-L1 after inoculation of colon cancer cells into mice resulted in significant anti-tumor effects, whereas the effects of monotherapy were not pronounced. This was due to the fact that combined blockade resulted in higher levels of cytokines (IFN-γ, TNF-α, and granzyme B) production by tumor-specific CD8^+^ T cells from tumor-draining lymph nodes compared to single blockade or control group 142. In addition, it was reported that anti-VISTA reduced the resistance to anti-PD-(L)1 therapy since anti-VISTA increased antigen presentation and the expression of IFN-regulated genes, reduced myeloid cell-mediated suppression, and simultaneously improved T cell infiltration 143. All in all, VISTA blockade exhibits synergistic anti-tumor effects with anti-PD-(L)1 treatment, and this might be due to that VISTA blockade improves T cell infiltration and reduces the resistance to anti-PD-(L)1 treatment via increasing the production of IFN-γ.

### IDO

Indoleamine 2,3-dioxygenase 1 (IDO1), as the most widely studied indoleamine 2,3-dioxygenase, is the primary rate-limiting enzyme of tryptophan metabolism through the kynurenine pathway, which may be dysregulated in various disease states 144. Through tryptophan depletion and kynurenine production, IDO plays a role in suppressing T cell response and maintaining immune tolerance in various pathological processes 145. IDO1 metabolizes tryptophan to kynurenine, which subsequently activates AhR, resulting in the upregulation of SOCS3. This, in turn, inhibited the activation of the JAK-STAT1 signaling pathway and reduced the secretion of CXCL9 and CXCL10, thereby decreasing T-cell infiltration 146. Moreover, the metabolism of tryptophan into kynurenine also promotes the expansion of Tregs, which inhibits inflammatory response by secreting anti-inflammatory cytokines, and suppresses the production of pro-inflammatory cytokines and the infiltration of neutrophils and Th1/Th17 cells 147.

In the field of cancer therapy, IDO1 has attracted more attention than IDO2. IDO1 is often silent in normal tissues but is expressed in restricted tissues 148. However, overexpression of IDO is observed in various tumors and is associated with poor prognosis 149, 150. Additionally, IFN-γ is believed to induce the expression of IDO 149.

It was found that the IDO1 inhibitor PF-06840003, when used in combination with anti-PD-L1, could induce a higher proportion of T cells secreting IFN-γ and the expression of the cytolytic enzyme granzyme A, thereby enhancing anti-tumor effects. Additionally, it was found that treatment with anti-CTLA-4 and anti-PD-L1 induced IDO1 expression, and this might be due to the secretion of IFN-γ by activated T cells after treatment, as IFN-γ was identified as an inducer of IDO1^151^. Similarly, another study found that treatment with PD-L1 blockade led to upregulation of IDO expression *in vivo*. The combination therapy of PD-L1 blockade and IDO inhibitor can improve anti-tumor effects by increasing the frequency of IL-2-producing and proliferating polyfunctional T cells within the tumor, and prolonging the duration and frequency of peripheral tumor-reactive lymphocytes at a later stage. However, the combination therapy did not increase the early anti-tumor CD8^+^ T cell counts in the tumor-draining lymph nodes 152. When combined with PD-L1 blockade, IDO nano-inhibitor enhanced the anti-tumor efficacy of anti-PD-L1 via decreasing the proportion of immune suppressive cells (Tregs) and increasing the proportion of immune effector cells (IFN-γ secreting tumor-infiltrating T cells) 153. Moreover, the co-expression of IDO-1 and PD-L1 might be related to the enhanced anti-tumor efficacy in NSCLC clinical trials with dual blocking of PD-1/PD-L1 and IDO-1^154^. To sum up, the combination of IDO1 inhibitor and anti-PD-L1 shows enhanced anti-tumor effects. This might be due to the fact that anti-PD-L1 induces IDO1 expression, then IDO1 inhibitor decreases proportion of Tregs and increases the proportion of immune effector cells.

### CD47

CD47, also known as integrin-associated protein (IAP), is often utilized by cancer cells to evade immune system surveillance and attack. CD47 is generally expressed in human tissues, but the mRNA levels vary across different tissues. Form 2 is commonly expressed in bone marrow-derived cells, endothelial cells, and fibroblasts, and form 4 is most commonly expressed in neural tissues 155. The CD47-SIRPα axis plays a role in tumor evasion in the process of phagocytosis mediated by macrophages, dendritic cells, and other phagocytic cells 156. It was found that SHP2 deneddylation, through the CD47/SIRPα axis, mediated tumor immune suppression in colon cancer, and the administration of allosteric SHP2 inhibitors sensitized immunotherapy-resistant colorectal cancer to immunotherapy 157. Moreover, CD47 could induce compartmental remodeling of tumor-infiltrating immune cells within the pancreatic cancer microenvironment 158. Blocking the CD47-SIRPα axis was considered to be a promising option for cancer treatment 156. In addition, CD47 could also bind to thrombospondin-1 (TSP-1), thereby limiting the production of H_2_S and inhibiting H_2_S-induced ERK1/2 phosphorylation, thus suppressing the activation of T cells 159.

Interestingly, PD-L1 and CD47 are co-expressed in various cancers 160. The dual blockade of CD47 and PD-1/PD-L1 shows synergistic anti-tumor effects. It was found that CD47 absence markedly improved anti-tumor efficacy mediated by anti-PD-1 therapy 86. Compared to PD-L1 blockade, CD47/PD-L1 bispecific antibody has superior anti-tumor efficacy, through increasing the levels of pro-inflammatory cytokines (IL-1β, IL-12, and IL-18), effector cytokines (IFN-γ) and T cell-recruiting chemokines (CXCL9, CXCL10, and CCL5), amplifying the systemic CD8^+^ T cell response, expanding the pool of intra-tumoral CD8^+^ T cells, reprogramming myeloid population, driving innate activation within the TME, increasing the frequency of stem-like progenitor and effector CD8^+^ T cells in the tumor, and promoting the differentiation of progenitor CD8^+^ T cells into an effector-like state^161^. Furthermore, CD47 and PD-L1 bispecific antibody (6MW3211) was found to exhibit lower toxicity reactions and synergistic anti-tumor effects via increasing IFN-γ levels and promoting phagocytosis of macrophages 160. In a study on B-cell lymphoma, dual blockade with anti-CD47 and anti-PD-L1 therapy activated CD8^+^ T cells, increased the secretion of perforin, granzyme B, and IFN-γ, and enhanced macrophage infiltration 162. To sum up, dual blockade of CD47 and PD-1/PD-L1 shows synergistic anti-tumor effects, and this might be due to the fact that CD47 is co-expressed with PD-L1 in various cancers, and CD47 blockade promotes phagocytosis of macrophages and activates CD8^+^ T cells.

### Adenosine A2AR

Adenosine A2A receptor (A2AR) is expressed in immune tissues and various immune cells. A2AR was initially identified as a critical and non-redundant negative regulatory factor that protected normal tissues from inflammatory damage. Later, it was found to play a role in shielding tumors from attack by anti-tumor T cells 163. The current perspective is that A2AR agonists regulate immune cells. For T cells, adenosine/A2AR can inhibit CD4^+^ T cells, increase the generation of Foxp3^+^ Tregs, and block the cytotoxic function of CD8^+^ T cells. For NK cells, the adenosine/A2AR signal limits the NK cell maturation and proliferation. For macrophages, adenosine/A2AR can induce the conversion of macrophages into the M2 phenotype, which promotes tumor growth 163, 164. Consequently, the blockade of A2AR or the absence of A2AR can lead to anti-tumor effects and the improvement of tumor-induced immunosuppression. Jenabian and colleagues found that A2AR increased intracellular cAMP levels in Jurkat cell line and CD4^+^ T cells, thereby inhibiting the demethylation of the IL-2 gene promoter region, and consequently suppressing the proliferation of CD4^+^ T cells and the production of IL-2 in CD4^+^ T cells 165. Additionally, it was also observed that A2AR antagonist could rescue tumor-reactive T cells (mainly CD8^+^ T cells) by reducing cAMP levels, freeing anti-tumor T cells from adenosine-mediated suppression, and enhancing the production of pro-inflammatory cytokines 166. In a clinical study on renal cell carcinoma, A2AR antagonists led to significant tumor regression, and longer disease control time was associated with CD8^+^ T cell infiltration into the TME 167.

The combination of A2AR blockade with anti-PD-1/PD-L1 can enhance the therapeutic effect. PD-1 blockade upregulated A2AR expression on CD8^+^ TILs, which might account for the dual blockade combination therapy being more effective. Additionally, the combination blockade of PD-1 and A2AR increased the production of IFN-γ by tumor-infiltrating CD8^+^ T cells both *in vitro* and *in vivo* 168. Furthermore, the primary tumors with higher PD-L1 expression and lower A2AR expression showed better outcomes and longer overall survival in patients treated with anti-PD-1 antibody alone or in combination with anti-PD-1 and anti-CTLA4; whereas increased expression of A2AR was associated with poor outcomes and short survival 169. CPI-444 (an A2AR antagonist) moderately suppressed tumor growth, while combined with anti-PD-1 showed significant anti-tumor efficacy *in vivo* (causing tumor regression and improving survival), which might be related to the inhibition of PD-1 and LAG-3 expression by CPI-444 on CD8^+^ T cells and Tregs 170.

In summary, the preclinical studies and clinical trials on PD-1/PD-L1 blockade combination with other ICBs in cancer therapy have been summarized in** Table 1A, B, C, D** and **Table 2A, B, C, D**, showing synergistic anti-tumor efficacy to some extent. However, the combinations often resulted in increased toxicity. For instance, in a clinical trial on metastatic melanoma, the rate of confirmed objective response was 61% (44 of 72 patients) in the group that received both ipilimumab and nivolumab (combination group) versus 11% (4 of 37 patients) in the group that received ipilimumab and placebo (ipilimumab-monotherapy group), with complete responses reported in 16 patients (22%) in the combination group and no patients in the ipilimumab-monotherapy group. Notably, the incidence of grade 3-4 drug-related adverse events was found to be 54.3% for combination therapy (nivolumab and ipilimumab) compared to ipilimumab monoblockade of 23.9% 171. Similarly, in another clinical trial (phase Ib) on resectable esophageal/gastroesophageal junction cancer, the incidence of grade 3 or higher treatment-related adverse events was 43.8% in the neoadjuvant nivolumab-relatlimab group compared to 18.8% in the nivolumab group 172. It has been suggested that immune checkpoint blockade-induced irAEs may correlate with their anti-tumor efficacy and may even serve as a marker of response to immune checkpoint blockade therapy 7, 33. Thus, it can be seen that the therapeutic potential of PD-1/PD-L1 blockade in combination with other ICBs, but the combinations often lead to an increase in the incidence of severe irAEs. More effective management of irAEs will be a prerequisite for the combined application of ICBs in clinical practice.

## PD-1/PD-L1 Blockade in Combination with Non-Immunotherapeutic Strategies

Besides, some preclinical studies and clinical trials confirmed that PD-1/PD-L1 blockade achieved synergetic anti-tumor treatment benefits when combined with some classical treatments. Here, we briefly discuss the combination with chemotherapy, radiotherapy, and targeted therapy.

### Combination with chemotherapy

Chemotherapy is the classical treatment for cancers, but systematic toxic adverse effects limit its application. It was found that PD-1/PD-L1 blockade achieved synergetic anti-tumor treatment benefits when combined with chemotherapy. For example, in mouse model of pancreatic cancer liver metastasis, the group treated with gemcitabine and anti-PD-1 had the lowest average volume of liver metastatic nodules and prolonged survival, compared to gemcitabine alone or anti-PD-1 alone. The mechanism may be related to the enhancement of immune responses mediated by Th1 lymphocytes and M1 macrophages, as well as CD8^+^ T cells 173. In a phase 3 clinical trial involving advanced gastric cancer, gastroesophageal junction cancer, and esophageal adenocarcinoma, the combination of nivolumab with chemotherapy showed superior overall survival (OS) and progression-free survival (PFS) benefits compared to chemotherapy alone, along with acceptable safety 174. Additionally, a retrospective cohort study on nodular-type oral mucosal melanoma revealed that patients treated with chemotherapy combined with anti-PD-1 showed significant improvements in 2-year OS and PFS, and were safer and better tolerated, when compared to chemotherapy alone 175.

### Combination with radiotherapy

Radiotherapy, as a first-line oncological therapy, plays the role of treatment, prevention of recurrence, and palliative care in different types of tumors. However, it is noted that radiotherapy may lead to the activation of immunosuppressive pathways in the TME by increasing TIL frequency, upregulating PD-L1 and MHC-I expression 176. Thus, radiotherapy may benefit immunotherapy 177. For example, in a systematic review and meta-analysis study on NSCLC, it was found that the combination therapy of PD-1/PD-L1 inhibitors and radiotherapy can improve the OS, PFS, and tumor response rate of advanced NSCLC patients without increasing serious adverse events 178. Similarly, in another study, the combination therapy of radiotherapy and anti-PD-1/PD-L1 treatment had enhanced anti-tumor efficacy. Radiotherapy made nasopharyngeal carcinoma cells sensitive to the cytotoxic effects of NK cells, and upregulated the expression of PD-L1 on nasopharyngeal carcinoma cells and PD-1 on NK cells. Blocking the PD-L1/PD-1 checkpoint further increased the cytotoxicity of NK cells against nasopharyngeal carcinoma cells during radiotherapy 179.

### Combination with targeted therapies

Targeted therapies, based on the unique molecular markers or signaling pathways in cancer cells, have achieved therapeutic effects in numerous cancers while minimized adverse effects on normal cells and tissues.

#### Epidermal growth factor receptor (EGFR)

In EGFR-mutant lung adenocarcinoma, the combination of EGFR inhibitor erlotinib and anti-PD-1 monoclonal antibody significantly inhibited tumor growth. However, this synergistic anti-tumor effect was not observed in EGFR wild-type tumors 180. In another research, it was found that after acquiring resistance to the EGFR inhibitor gefitinib, the expression of PD-L1 in the specimens increased from less than 1% to 50% or more. This suggested that patients would be more likely to benefit from anti-PD-1 after EGFR inhibitor treatment, providing a rationale for the combination of EGFR-targeted drugs and PD-1/PD-L1 blockades 181.

#### Human epidermal growth factor receptor-2 (HER2)

HER2-positive is identified by the overexpression of the HER2 receptor due to HER2/ERBB2 gene amplification. It was confirmed that HER2-targeted therapy significantly improved post-treatment disease-free survival of HER2-positive breast cancer patients 182. However, longtime treatment showed unsatisfactory response rate, development of drug resistance, and disease recurrence 183. Combination therapy of anti-PD-1/PD-L1 (BMS-202) and trastuzumab (a drug targeting HER2) significantly reduced the survival rate and invasiveness of breast cancer cells 184. In a clinical trial on HER2-positive gastric cancer, the addition of pembrolizumab to the standard treatment (trastuzumab and chemotherapy) achieved significant improvements, reducing tumor size and increasing the objective response rate 185.

#### Vascular endothelial growth factor (VEGF)

The abnormal tumor vasculature system may mediate immune suppression in the TME. The combination of VEGF-target drugs and anti-PD-1/PD-L1 had shown promise and potential effects 186. In mouse model of small cell lung cancer, it was found that the combination of anti-VEGF and anti-PD-L1 had a synergistic effect, characterized by improved PFS, OS, and enhanced CD4^+^ T cell infiltration in the tumor 187. In another study, the dual blockade of VEGFR-2/PD-1 significantly delayed tumor growth in mice with liver cancer. The potential mechanisms of combination therapy include reprogramming of the TME (such as a significant increase in the number of tumor-infiltrating CTLs, and upregulation of PD-L1 and PD-1 expression following VEGFR-2 blockade 188.

#### Poly-ADP-ribose polymerase (PARP)

PARP inhibitors, a class of drugs targeting poly-ADP-ribose polymerase, are often used to treat cancer patients with BRCA1 and BRCA2 gene mutations. In breast cancer, the PARP inhibitor significantly upregulated the expression of PD-L1 in cancer cells and in mouse model through the inactivation of GSK3β. Administering anti-PD-L1 treatment could restore the reduction of tumor-infiltrating cytotoxic CD8^+^ T cells after PARP inhibitor treatment 189. In ovarian cancer, the PARP inhibitor significantly upregulated PD-L1 expression through the Chk1 pathway *in vitro*, and treatment with anti-PD-L1 could reverse the suppression of CD8^+^ T cells caused by PARP inhibitor treatment. Furthermore, combined anti-PD-L1 with PARP inhibitor treatment showed a synergistic anti-tumor efficacy *in vivo* 190.

Based on the above, anti-PD-1/PD-L1 therapy showed better response to some extent when combined with chemotherapy, radiotherapy, or targeted therapies. However, the combinations couldn't lead to a reduction in the incidence of treatment-related adverse events. In a phase 3 trial clinical study on gastric, gastro-oesophageal junction, and oesophageal adenocarcinoma, it was found that 59% of the nivolumab-plus-chemotherapy group experienced grade 3-4 treatment-related adverse events, compared with 44% of the chemotherapy group 174. In another clinical trial (phase 3), pembrolizumab plus chemotherapy was superior to placebo plus chemotherapy for progression-free survival in patients with oesophageal squamous cell carcinoma (6.3 months vs 5.8 months). However, 266 (72%) patients in the pembrolizumab plus chemotherapy group experienced treatment-related adverse events of grade 3 or higher, while 250 (68%) patients in the placebo plus chemotherapy group 191. In addition, in a phase 2 clinical trial of platinum-resistant recurrent or metastatic nasopharyngeal carcinoma, the objective response rate was significantly higher in the bevacizumab and pembrolizumab group (58.3% [95% confidence interval: 36.6-77.9] than that in the pembrolizumab group (12.5% [2.7-32.4], and the grade 3 treatment-related adverse events occurred in 29% (7/24) of the combination group compared to 8% (2/24) in the pembrolizumab group 192. It can be seen that the therapeutic potential of PD-1/PD-L1 blockade in combination with non-immunotherapeutic strategies. However, the treatment-related adverse events need to be addressed, such as irAEs induced by PD-1/PD-L1 blockade, systematic toxicity induced by chemotherapy, local dermatitis induced by radiotherapy, etc.

## Conclusion and Prospects

In recent years, despite the rapid development of ICBs in the field of tumor treatment, their clinical use is limited by low response rates and the potential problem of drug resistance. Therefore, it is urgent to expand the population that can benefit from ICBs and overcome resistance. Among them, the PD-1/PD-L1 is the most widely studied and promising immune checkpoint in cancer immunotherapy research and clinical application. Thus, it is important and meaningful to investigate the complex mechanisms of PD-1/PD-L1 blockade resistance. In this review, we summarized the vital aspects. However, it should be recognized that resistance mechanisms are dynamic and complex due to the high heterogeneity of patients and tumors. In the current review, we focused on the strategies of combining PD-1/PD-L1 blockade with other ICBs, which showed synergetic anti-tumor effects in preclinical and clinical studies.

For the combination therapy of PD-1/PD-L1 and other ICBs, some issues still need to be resolved and studied: (1) To uncover more targets and more effective immune checkpoint combinations based on preclinical and clinical research; (2) Personalized cancer immunotherapy treatment plans need to be formulated based on tumor heterogeneities, drug resistance issues, and individual immune status; (3) Further research on biomarkers for combination therapy is needed, such as discovering more effective biomarkers, combining multiple biomarkers, developing non-invasive biomarker detection, etc., to predict treatment response and adverse reactions more predictively; (4) Preclinical research related to combination therapy needs more precise research for clinical translation, such as tumor organoid culture can simulate the TME and thus better predict drug sensitivity and assess prognosis; (5) Combination therapy plans still need to be optimized from multiple aspects such as efficacy, safety, avoiding and handling immune-related adverse events.

## Figures and Tables

**Table 1A:** The preclinical studies of the combination of PD-1/PD-L1 blockade with other ICBs in cancer therapy

Targets	PD-1/PD-L1 blockade	Other ICB	Tumor types	Model	Findings (mechanisms)	References	

PD-L1×CTLA-4	Anti-PD-L1 antibody	Anti-CTLA-4 antibody	Colon Cancer	BALB/c mice with CT26 orthotopic Colon Cancer	Dual CTLA-4 and PD-L1 blockade increased CD4^+^ and CD8^+^ T cells significantly in the tumors, and decreased intra-tumoral Tregs.	108	
PD-1/PD-L1×CTLA-4	Anti-PD-1 (RMP1-14); anti-PD-L1 (9G2)	Anti-CTLA-4 (9D9)	Melanoma	C57BL/6 mice with B16 Melanoma	Dual CTLA-4 and PD-1 blockade enhanced the CD8^+^ T cells/Tregs and CD8^+^ T cells/MDSCs ratios in melanoma mice. The elevated Teff appeared to correlate with high levels of inflammatory cytokines like IFN-γ and TNF-α in the tumor tissues.	114	
PD-1/PD-L1×CTLA-4	Anti-PD-1/αPD-L1	Anti-CTLA-4	Colon carcinoma; ovary cancer	BALB/c or C57BL/6 mice bearing with colon carcinoma cell line CT26 or ovarian carcinoma cell line ID8-VEGF.	Dual blockades of PD-1 and CTLA-4 increased IFN-γ production, reduced the secretion of TGF-β and IL-10, and promoted TIL proliferation and its cytolytic function through upregulation of ribosomal S6 kinase, T-bet, and Eomes.	115	

**Table 1B:** The preclinical studies of the combination of PD-1/PD-L1 blockade with other ICBs in cancer therapy

	PD-1/PD-L1 blockade	Other ICB	Tumor types	Model	Findings (mechanisms)	References
PD-1×TIM-3	PD-1-blocking antibody	TIM-3-blocking antibody	Lung adenocarcinoma	Genetically engineered mouse models of lung cancer: EGFR L858R T790M mutation and CC10 RTTA double-positive mice, KrasG12D mice.	TIM-3 blockade treatment after PD-1 blockade failure significantly improved anti-tumor efficacy and survival. The combination therapy increased IFN-γ production and proliferation of TIM-3^+^ CD8^+^ T cells from PD-1-resistant mice, decreased the expression of some tumor-promoting cytokines, such as IL-6 and progranulin.	91
PD-1×TIM-3	Anti-PD1 mAb	Anti-TIM-3 mAb	Hepatocellular carcinoma	BALB/c nude mice bearing with HepG2 cells	The combined blocking exhibited a more significant anti-tumor effect than a single blockade in mice with hepatocellular carcinoma, through increased production of T cell effector cytokines (IFN-γ and TNF-α) and TILs, decreased levels of immunosuppressive cytokines (IL-10 and IL-6) and the amounts of PD-1^+^TIM-3^+^CD8^+^ T cells within the TME.	124
PD-1×TIGIT	Anti-PD-1 mAb	Anti-TIGIT mAb 10D7.G8	Melanoma	CD8^+^ T lymphocytes from PBMCs obtained from patients	Dual blockades of TIGIT and PD-1 further increased NY-ESO-1-specific CD8^+^ T cell counts. PD-1 blockade increased TIGIT expression, but TIGIT blockade could not increase PD-1 expression.	136
PD-1×TIGIT	Anti-PD-1 (4 H2)	Anti-TIGIT (clone 4B1 mIgG2 a, depleting isotype)	Glioblastoma	C57 BL/6 J mice with intracranial tumor	Both combined blockade and PD-1 single blockade can establish anti-tumor immune memory. The superior efficacy of combination therapy may be due to increased CD8^+^ and CD4^+^ T-cell infiltration, higher IFN-γ and TNF-α production, and reduced tumor-infiltrating dendritic cells.	137

**Table 1C:** The preclinical studies of the combination of PD-1/PD-L1 blockade with other ICBs in cancer therapy

Targets	PD-1/PD-L1 blockade	Other ICB	Tumor types	Model	Findings (mechanisms)	References
PD-L1×VISTA	Anti-PD-L1 mAb	Anti-VISTA mAb	Colon cancer	C57BL/6 mice with CT26 colon cancer	Combined treatment showed significant anti-tumor effects in mice with colon cancer, through increased cytokine (IFN-γ, TNF-α, and granzyme B) production by tumor-specific CD8^+^ T cells from tumor-draining lymph nodes more than single blockade or control.	142
PD-L1×IDO1	Anti-PD-L1 (clone 10F.9G2)	IDO1 inhibitor (PF-06840003)	Colon carcinoma	BALB/c and C57BL/6 mice with colon carcinoma	IDO1 inhibitor PF-06840003, when used in combination with anti-PD-L1, could induce a higher proportion of T cells secreting IFN-γ and the expression of the cytolytic enzyme granzyme A, thereby enhancing anti-tumor effects.	151
PD-L1×IDO	Anti-PD-L1 antibody (clone 10 F.9G2)	IDO inhibitor (INCB23843)	Melanoma	C57BL/6 mice bearing with B16-dsRed-SIY cells	Combination therapy increased IL-2-producing, proliferating polyfunctional T cells in the tumor, prolonged peripheral tumor-reactive lymphocytes' duration and frequency later on, but didn't boost early anti-tumor CD8^+^ T cells in tumor-draining lymph nodes.	152
PD-L1×IDO	Anti-PD-L1	NLG-RGD NI	Pancreatic cancer	pancreatic cancer cell line Pan02	IDO nano-inhibitor enhanced the anti-tumor efficacy of anti-PD-L1 via decreasing the proportion of Tregs and increasing the proportion of the IFN-γ secreting tumor-infiltrating T cells.	153

**Table 1D:** The preclinical studies of the combination of PD-1/PD-L1 blockade with other ICBs in cancer therapy

Targets	PD-1/PD-L1 blockade	Other ICB	Tumor types	Model	Findings (mechanisms)	References
PD-L1×CD47	IBI322 (CD47/PD-L1 bispecific antibody)	Burkitt's lymphoma, melanoma		NOD-SCID mouse bearing with Raji-PDL1 cell or A375 cells	CD47 and PD-L1 bispecific antibody (IBI322) was found to exhibit lower toxicity reactions and synergistic anti-tumor effects via increasing IFN-γ levels and promoting phagocytosis of macrophages.	193
PD-L1×CD47	Anti-PD-L1 mAb	SIRPα-Fc	B-cell lymphoma	BALB/c mice with lymphoma	Dual blockade with anti-CD47 and anti-PD-L1 therapy activated CD8^+^ T cells, increased the secretion of perforin, granzyme B and IFN-γ, and enhanced macrophage infiltration.	162
PD-1×A2AR	RMP1-14 (anti-PD-1 mAb)	SCH58261	Breast carcinoma	C57BL/6 and BALB/C mice bearing with MC38 cells and 4T1.2 cells	Blocking PD-1 upregulated A2AR expression on CD8^+^ TILs. The combination blockade of PD-1 and A2AR increased the production of IFN-γ by tumor-infiltrating CD8^+^ T cells both *in vitro* and *in vivo*.	168
PD-1×A2AR	Anti-PD-1 mAb (RMP1-14, Bioxcell)	CPI-444 (A2AR antagonist)	Colon cancer, melanoma	C57BL/6 mice bearing with MC38 cells and B16-OVA cells	CPI-444 moderately suppressed tumor growth, while combined with anti-PD-1 showed significant anti-tumor efficacy *in vivo* (causing tumor regression and improving survival), which might be related to the inhibition of PD-1 and LAG-3 expression by CPI-444 on CD8^+^ T cells and Tregs.	170

**Table 2A:** The clinical trials of the combination of PD-1/PD-L1 blockade with Other ICB in cancer therapy

Targets	PD-1/PD-L1 blockade	Other ICB	Clinicaltrial no.	Phase	Tumor types	Findings	References
PD-1×CTLA-4	Nivolumab	Ipilimumab	NCT03033576	II	Refractory metastatic melanoma	The combination of nivolumab and ipilimumab resulted in a statistically significant improvement in PFS over ipilimumab (hazard ratio, 0.63, p = 0.04). ORR were 28% and 9%, respectively (p = 0.05).	194
PD-1×CTLA-4	Nivolumab	Ipilimumab	NCT02716272	II	Relapsed malignant pleural mesothelioma	In the nivolumab group, 24 (44%) of 54 patients achieved 12-week disease control, compared to 27 (50%) of 54 in the combination group. In the intention-to-treat population, 25 (40%) of 63 in the nivolumab group and 32 (52%) of 62 in the combination group achieved 12-week disease control.	195
PD-1×CTLA-4	Nivolumab	Ipilimumab	NCT01844505	III	Melanoma	Median PFS was 11.5 months for combined treatment (2.9 months for ipilimumab alone, 6.9 months for nivolumab alone). In PD-L1-positive patients, median PFS was 14.0 months for both combination and nivolumab alone groups. In PD-L1-negative patients, combination PFS was 11.2 months vs 5.3 months with nivolumab alone.	196
PD-L1×CTLA-4	Durvalumab	Tremelimumab	NCT02592551	II	Malignant pleural mesothelioma	Patients receiving combination blockades had longer median overall survival compared with those receiving monotherapy. Tumor PR occurred in 6 of 17 patients receiving ICB and thoracotomy (35.3%), among which major PR (>90% tumor regression) occurred in 2 (11.8%).	197
PD-L1×CTLA-4	Durvalumab	Tremelimumab	NCT02319044	II	Recurrent or metastatic HNSCC	Objective response rate was 7.8% in the combination arm (n = 129), 9.2% for durvalumab monotherapy (n = 65), and 1.6% for tremelimumab monotherapy (n = 63); median overall survival for all patients treated was 7.6, 6.0, and 5.5 months, respectively.	198
PD-1×CTLA-4	Cadonilimab		NCT04220307	II	Recurrent or metastatic nasopharyngeal carcinoma	ORR was 26.1 %. The ORR were 44.4 % and 14.3 % in patients with tumor PD-L1 expression ≥50 % and <50 %, respectively. ORR was achieved in 40.0 % of patients with EBV-DNA level <4000 IU/ml and 15.4 % of those with ≥4000 IU/ml.	199

ICB: immune checkpoint blockades; ORR: objective response rate; DCR: disease control rate; PFS: progression-free survival; PR: partial response; HNSCC: head and neck squamous cell carcinoma.

**Table 2B:** The clinical trials of the combination of PD-1/PD-L1 blockade with other ICBs in cancer therapy

Targets	PD-1/PD-L1 blockade	Other ICB	Clinicaltrial no.	Phase	Tumor types	Findings	References
PD-L1×TIM-3	LY3300054	LY3321367	NCT02791334	Ib	Microsatellite instability-high/MMR-deficient tumors	Objective response occurred in 13 patients (32.5%) with monotherapy, 9 (45.0%) in the PD-1/PD-L1 inhibitor-naïve combination cohort, and 1 patient (4.5%) in the PD-1/PD-L1 inhibitor-resistant/refractory combination cohort.	200
PD-L1×TIM-3	LY300054	LY3321367	NCT03099109	Ia/b	Advanced solid Tumors	In the NSCLC monotherapy expansion cohort, anti-PD-1/L1 refractory patients (n= 23, ORR 0%, DCR 35%, PFS 1.9 months) versus anti-PD-1/L1 responders (n = 14, ORR 7%, DCR 50%, PFS 7.3 months). In combination expansion cohorts (n = 91), ORR and DCR were 4% and 42%.	201
PD-1×TIM-3	Spartalizumab	Sabatolimab	NCT02608268	I/Ib	Advanced solid Tumors	No response was seen with sabatolimab. 5 patients receiving combination treatment had PR (6%; lasting 12-27 months) in colorectal cancer (n = 2), NSCLC, malignant perianal melanoma, and SCLC.	202
PD-L1×TIM-3	LY3415244		NCT03752177	I	Advanced solid Tumors	One patient with PD-1 refractory NSCLC had a near partial response (29.6%).	203

ICB: immune checkpoint blockades; ORR: objective response rate; DCR: disease control rate; PFS: progression-free survival; NSCLC: non-small cell lung cancer; CAR-T: chimeric antigen receptor T-cell immunotherapy.

**Table 2C:** The clinical trials of the combination of PD-1/PD-L1 blockade with other ICBs in cancer therapy

Targets	PD-1/PD-L1 blockade	Other ICB	Clinicaltrial no.	Phase	Tumor types	Findings	References
PD-1×LAG-3	Nivolumab	Relatlimab	NCT04205552	II	Resectable NSCLC	Major pathological and objective radiographic responses were achieved in 27% and 10% (nivolumab) and in 30% and 27% (nivolumab and relatlimab) of patients, respectively. With 12 months median duration of follow-up, disease-free survival and overall survival rates at 12 months were 89% and 93% (nivolumab), and 93% and 100% (nivolumab and relatlimab).	204
PD-1×LAG-3	Nivolumab	Relatlimab	NCT03470922	II/III	Untreated advanced melanoma	The median PFS was 10.1 months; PFS at 12 months was 47.7%.	205
PD-1×LAG-3	Spartalizumab	Ieramilimab	NCT02460224	I/II	Advanced malignancies	Anti-tumor activity was observed in the combination arm, with 3 (2%) complete response and 10 (8%) partial response in a mixed population of tumor types. In the combination arm, eight patients (6.6%) experienced stable disease for 6 months or longer versus six patients (4.5%) in the single-agent arm.	206
PD-1×LAG-3	Tebotelimab		NCT03219268	I	Solid tumors and hematologic cancers	There were tumor decreases in 34% (59/172) of response-evaluable patients in the dose-escalation cohorts, with objective response in multiple solid tumor types, including PD-1-refractory disease, and in LAG-3 non-Hodgkin lymphomas, including CAR-T refractory disease.	207

ICB: immune checkpoint blockades; ORR: objective response rate; DCR: disease control rate; PFS: progression-free survival; NSCLC: non-small cell lung cancer; CAR-T: chimeric antigen receptor T-cell immunotherapy.

**Table 2D:** The clinical trials of the combination of PD-1/PD-L1 blockade with other ICBs in cancer therapy

Targets	PD-1/PD-L1 blockade	Other ICB	Clinicaltrial no.	Phase	Tumor types	Findings	References
PD-1×TIGIT	Pembrolizumab	Vibostolimab	NCT02964013	I	Advanced solid tumors, including NSCLC	Part A: confirmed ORR was 0% with monotherapy and 7% with combination therapy. Part B: confirmed ORR was 3% with monotherapy and 3% with combination therapy.	208
PD-L1×TIGIT	Atezolizumab	Tiragolumab	NCT03563716	II	PD-L1-positive NSCLC	21/67 patients (31.3%) in the combined group versus 11/68 patients (16.2%) in the placebo plus atezolizumab group had an objective response. Median PFS was 5.4 months in the combined group versus 3.6 months in the placebo plus atezolizumab group.	209
PD-1×IDO	Pembrolizumab	Epacadostat	NCT03414229	II	Advanced sarcoma	The best ORR at 24 weeks was 3.3% (PR, 1/30). The median PFS was 7.6 weeks. Combined treatment was well tolerated and showed limited antitumor activity in sarcoma.	210
PD-1×IDO	Pembrolizumab	Epacadostat	NCT02752074	III	Unresectable stage III or IV melanoma	No significant differences were found between the treatment groups for PFS (median 4.7 months for epacadostat plus pembrolizumab vs 4.9 months for placebo plus pembrolizumab) or overall survival.	211
PD-1×CD47	Pembrolizumab	Evorpacept	NCT03013218	I	Advanced solid tumours	Among patients who received evorpacept plus pembrolizumab, overall responses were recorded in 4/20 patients with HNSCC, in 1/ 20 patients with NSCLC, and in 4/19 patients with gastric or gastroesophageal junction cancer.	212
PD-L1×A2AR	Durvalumab	AZD4635	NCT02740985	Ia/b	Solid tumors	In patients with metastatic castration-resistant prostate cancer receiving monotherapy or combination treatment, tumor responses (2/39 and 6/37, respectively) and prostate-specific antigen responses (3/60 and 10/45, respectively) were observed. High versus low blood-based adenosine signature was associated with median PFS of 21 weeks versus 8.7 weeks.	213

ICB: immune checkpoint blockades; ORR: objective response rate; DCR: disease control rate; PFS: progression-free survival; PR: partial response; NSCLC: non-small cell lung cancer; HNSCC: head and neck squamous cell carcinoma.

## Abbreviations

PD-1: programmed death-1
PD-L1: programmed death-ligand 1
TME: tumor microenvironment
ICBs: immune checkpoints blockades
ITIM: immune receptor tyrosine-based inhibitory motif
ITSM: immune receptor inhibitory tyrosine-based switch motif
TCR: T cell receptor
irAEs: immune-related adverse events
SD: stable disease period
TILs: tumor-infiltrating lymphocytes
CAFs: cancer-associated fibroblasts
MDSCs: myeloid-derived suppressor cells
TAMs: tumor-associated macrophages
CA-MSCs: cancer-associated mesenchymal stem cells
MHC: major histocompatibility complex
ECM: extracellular matrix
LSD1: lysine-specific demethylase 1
NSCLC: non-small cell lung cancer
APCs: antigen-presenting cells
TMB: tumor mutation burden
MMR: mismatch repair
MSI: microsatellite instability
Tregs: regulatory T cells
CTLs: cytotoxic T lymphocytes
FMT: fecal microbiota transplantation
CTLA-4: cytotoxic T-lymphocyte associated antigen-4
TIM-3: T cell immunoglobulin-3
LAG-3: lymphocyte activation gene 3
TALs: tumor-associated lymphocytes
TIGIT: T cell immunoreceptor with immunoglobulin and ITIM domain
VISTA: V-domain Ig suppressor of T cell activation
IDO: indoleamine 2,3-dioxygenase
A2AR: adenosine A2A receptor
TSP-1: thrombospondin-1
OS: overall survival
PFS: progression-free survival
EGFR: epidermal growth factor receptor
HER2: human epidermal growth factor receptor-2
PARP: poly-ADP-ribose polymerase
VEGF: vascular endothelial growth factor
